# Antimicrobial Resistance in Fecal *Escherichia coli* from Humans and Pigs at Farms at Different Levels of Intensification

**DOI:** 10.3390/antibiotics9100662

**Published:** 2020-09-30

**Authors:** Kamonwan Lunha, Thongpan Leangapichart, Jatesada Jiwakanon, Sunpetch Angkititrakul, Marianne Sunde, Josef D. Järhult, Gunilla Ström Hallenberg, Rachel A. Hickman, Thomas Van Boeckel, Ulf Magnusson

**Affiliations:** 1 Department of Clinical Sciences, Swedish University of Agricultural Sciences, SE-750 07 Uppsala, Sweden; kmnwn.lunha@gmail.com (K.L.); gunilla.strom.hallenberg@folkhalsomyndigheten.se (G.S.H.); 2 Section for Food Safety and AMR, Norwegian Veterinary Institute, N-0106 Oslo, Norway; Thongpan.Leangapichart@vetinst.no (T.L.); marianne.sunde@vetinst.no (M.S.); 3 Research Group for Animal Health Technology, Khon Kaen University, Khon Kaen 40002, Thailand; jatgiw@kku.ac.th (J.J.); Sunpetch@kku.ac.th (S.A.); 4 Department of Medical Sciences, Uppsala University, SE-751 85 Uppsala, Sweden; josef.jarhult@medsci.uu.se; 5 Department of Biomedical Chemistry and Microbiology, Uppsala University, SE-752 37 Uppsala, Sweden; rachel.hickman@medsci.uu.se; 6 Institute for Environmental Decisions, ETH, 8092 Zurich, Switzerland; thomas.vanboeckel@env.ethz.ch; 7 Center for Diseases Dynamics Economics and Policy, Washington, DC 20005, USA

**Keywords:** antimicrobial resistance, pigs, farmers, Thailand, farming intensification

## Abstract

The overall aim of the current study was to test the hypotheses that (i) antibiotic resistance in bacteria were more frequent in clinically health pigs in intensified company owned, medium-scale farms (MSFs) (100–500 sows) than in pigs in family-owned, small-scale farms (SSFs) (1–50 sows) and (ii) that farmers working at the MSFs were more prone to attain antibiotic resistant bacteria than farmers working at SSFs. The study was conducted in North-Eastern Thailand, comprising fecal *Escherichia coli* isolates from pigs, farmers working with the pigs (contact humans) and persons living in the same household as the farmer (non-contact humans) at 51 MSFs and 113 SSFs. Samples from all farms were also screened for methicillin-resistant staphylococcus aureus (MRSA), which was not detected in pig samples, but was found in one human sample. Susceptibility was tested by disc-diffusion for seven antibiotics commonly used in the study area. Resistance in pig isolates from MSFs were more frequent for chloramphenicol which (*P* < 0.001), trimethoprim/sulfamethoxazole (*P* < 0.001) and gentamicin (*P* < 0.05) compared with isolates from SSFs, whereas the opposite was true for tetracycline (*P* < 0.01). Resistance in the human isolates was lower than those in the isolates from pigs for tetracycline, trimethoprim/sulfamethoxazole and chloramphenicol (*P* < 0.001). The frequency of resistance in the contact human samples from SSFs and MSFs did not differ. There was no difference between isolates from contact and non-contact humans for any of the tested antibiotics. Multidrug resistance in isolates from pigs was 26%, significantly higher (*P* < 0.01) than the 13% from humans. The data indicate that (i) resistance to antibiotics, including those critical and highly important for human medicine, were more common in fecal *E. coli* from pigs at the MSFs than at the SSFs, whereas (ii) the resistance in fecal *E. coli* from pig farmers seemed not to be influenced by the level of intensification of the farm they were working at.

## 1. Introduction

Antimicrobial resistance (AMR) is a growing global public health threat. The use of antimicrobial agents for more than seven decades in human and veterinary medicine has contributed to the selection and dissemination of antimicrobial-resistant bacteria in animals, humans and the environment [1]. Antimicrobials are extensively used in food-producing animal breeding to cure animals when sick as well as on regular bases to prevent disease and, in some parts of the world, to promote growth [2,3]. However, the latter kind of antimicrobial use is being continuously phased out and is now banned in many countries [4]. Still, large amounts of antibiotics are being used, in particular in poultry and pig farming in many places around the world. When transforming poultry or pig farming from small family subsistence farming to larger commercial farms with high demand on productivity, the regular use of antibiotics is often regarded as an easy method to maintain healthy and productive animals instead of applying antibiotic-free disease prevention methods [5]. This kind of transformation of the pig sector from small-scale to commercial medium- or large-scales is prominent in South East Asia [6], making the region an interesting place to study the emergence of AMR in livestock breeding in relation to farming systems. Additionally, in South-East Asia, antimicrobials are freely available over the counter for use in both humans and animals, therefore likely contributing to the extensive use in the livestock sector and the widely occurring AMR [7,8,9]. For instance, in Thailand, an extensive use of antimicrobials in the livestock sector has been documented, including agents considered to be critical for human medicine, such as aminoglycosides, fluoroquinolones and macrolides [2,10,11]. As a result, high levels of AMR in zoonotic bacteria, such as *Salmonella* and *Campylobacter* spp., have been reported along the animal-food production system [12,13].

The fact that farm animals are potential reservoirs for antibiotic resistant bacteria [14,15] should place animal farm workers in a risk-group for attaining resistant bacteria from farm animals. However, reports on antibiotic resistance of microorganisms isolated from animal farm-workers are scanty, especially from low- and middle-income countries.

There are different approaches to assess the occurrence or prevalence of AMR in human or livestock populations. Commensal *Escherichia coli*, an abundant enteric bacterium, can serve as an indicator strain of AMR in the food chain as it is found in many compartments of the biota and do easily acquire AMR commonly found in the bacteria of different animal species. Therefore, *E. coli* may serve as an indicator for AMR in livestock as well as humans for investigating dynamic population changes in AMR [15] or for being used in AMR monitoring programs [16]. Another approach is to study the resistance in particular pathogenic bacteria of interest or relevance. One such bacteria is methicillin resistant *Staphylococcus aureus* (MRSA) known to be bi-directionally transferred between pigs and humans [17].

Here we present data from the Khon Kaen province in Thailand on the occurrence of antibiotic resistance in fecal *E. coli* isolates from healthy pigs in different farming systems, from healthy animal farm workers and from healthy individuals living in the same household but without contact with the pigs. Additionally, we present data on the occurrence of MRSA in the same groups of pigs and humans as well as in the farm environment. As the extensive use of antibiotics in many intensive animal farming systems may drive the development of antimicrobial resistance, we hypothesized that we would find a higher rate of AMR in the fecal *E. coli* isolated from pigs in intensified company owned medium-scale farms (MSFs) than in those from family-owned small-scale farms (SSFs). We therefore also hypothesized that, in the isolates from pig farmers at MSFs, we would find a higher rate of antibiotic resistance than in those from pig farmers working at SSFs.

## 2. Results

### 2.1. Occurrence of E. coli in Sample Population

From the total of 1111 fecal samples, 987 (89%) *E. coli* isolates were successfully isolated by antibiotic-free isolation methods to later screen the random *E. coli* population for antimicrobial susceptibility to seven antibiotic drugs. Most of them were pig isolates (757 isolates/839 fecal samples; 90%), of which 457/509 (90%) and 300/330 (91%) isolates were obtained from the intensified, company owned MSFs and family owned SSFs, respectively. For humans, *E. coli* were isolated from 88% (139/158) of the contact human fecal samples and from 80% (91/114) of the non-contact human samples.

### 2.2. Occurrence of Methicillin-Resistant Staphylococcus Aureus (MRSA)

One MRSA positive sample was obtained. This sample originated from a non-contact human from an MSF farm.

### 2.3. Antimicrobial Resistance in E. coli Isolates

Taking the porcine and human isolates together, 70% were resistant to at least one of the antibiotics tested (Table 1). On the other hand, 24% and 50% of pig and human isolates, respectively, were susceptible to all antibiotics tested for. Resistance to tetracycline (54%) was most prevalent, followed by trimethoprim/sulfamethoxazole (37%) and chloramphenicol (31%) resistance, while resistance to cefotaxime was only 1%. Resistance to meropenem was not detected.

Of the *E. coli* isolates from SSF pigs, 75% showed resistance to at least one of the antibiotics tested for (Table 1). Resistance to tetracycline (65%), trimethoprim/sulfamethoxazole (33%) and chloramphenicol (26%) was most common. (Table 1). The same rank-order was true for the MSF isolates, 53, 46 and 43%, respectively. Resistance in isolates from MSFs were significantly more frequently observed to chloramphenicol (*P* < 0.001), trimethoprim/sulfamethoxazole (*P* < 0.001) and gentamicin (*P* < 0.05), compared with isolates from SSFs. On the contrary, SSF isolates showed a higher frequency of tetracycline resistance than MSF isolates (*P* < 0.01).

Overall, 50% of the 230 *E. coli* isolates from human samples were resistant to at least one of the antibiotics tested for (Table 1). Resistance was most common to tetracycline followed by trimethoprim/sulfamethoxazole and chloramphenicol in isolates from both contact and non-contact humans. No significant difference was observed between isolates from contact and non-contact humans for any of the tested antibiotics. When comparing the frequency of resistance in the contact human samples from the SSFs with those from the MSFs, there was no difference (Table 2).

The frequency of AMR in the human isolates were lower than in the isolates from pigs for tetracycline, trimethoprim/sulfamethoxazole and chloramphenicol (*P* < 0.001).

### 2.4. Antimicrobial Resistance Profiles and Distribution

Overall, 23% of the isolates were multi drug resistant (MDR), with the highest frequency in isolates from MSF-pigs, followed by SSF-pigs and equal frequency for non-contact humans and contact humans (Table 1). Multidrug resistance was more common (*P* < 0.01) in isolates from pigs compared to isolates from humans. The most frequently observed MDR pattern common for all four categories of isolates was resistance to CHL-TET-SXT (18.8%, 15.6%, 5.8% and 6.6% of MSF-Pig, SSF-Pig, contact humans and non-contact humans, respectively). There were two isolates, from one MSF-pig and one contact human, resistant to all agents tested except meropenem.

There were 36 different antibiograms (patterns of antibiotic resistance) observed in this study. The number of different antibiograms was higher in the isolates from MSF and SSF pigs (27 and 22, respectively), than in human isolates (17 and 16 from contact and non-contact humans, respectively) (Table 3). Fourteen antibiograms were shared by the isolates from pigs and humans. The three most common antibiograms among the four categories of isolates were (i) in MSF-pig isolates, CHL-SXT-TET, TET and CHL-SXT; (ii) in SSF-pig isolates, TET, CHL-SXT-TET and SXT-TET; (iii) in contact human isolates, TET, CHL-SXT-TET, CHL-TET and SXT-TET; in non-contact human isolates, TET, SXT-TET and CHL-SXT-TET were most common.

### 2.5. Correlation among Resistance in the Isolates

The pairwise correlation analysis revealed varying degrees of correlation between the resistance to the different antibiotics tested for (Table 4). The strongest correlation was found between the pair chloramphenicol and trimetroprim-sulfametoxazole (Pearsons’ correlation, 0.60). Multi drug resistance was most commonly correlated with resistance to chloramphenicol, trimetroprim-sulfametoxazole, and tetracycline.

## 3. Discussion

In the current study conducted in Norther Eastern Thailand, the antibiotic resistance in fecal *E. coli* isolates collected from pigs and humans was high: 75% of the pig samples and 50% of the human samples were resistant to at least one of the antibiotics tested for. The observations in pigs are in accordance with previous studies from South-East Asia [7,10], but well above the prevalence reported from regions with low AMR prevalence, such as Northern Europe [18,19]. Only one quarter and half of pig and human isolates, respectively, were susceptible to all antibiotics tested for. The frequencies of resistance to the various antibiotics tested for, including MDR, were generally higher in isolates from pigs than from humans. Moreover, there were no differences in resistance frequencies between the farmers working with pigs and those living in the same household but not working with pigs. Interestingly, there were no differences in resistance frequencies between isolates from farmers working with pigs in SSFs and in MSFs, respectively, despite differences in AMR frequencies in isolates from pigs from these categories of farms. It should be noted, though, that the design of this study does not allow us to draw any firm conclusions regarding the transmission path of resistant bacteria. For such conclusions, a detailed transmission study using molecular tools is required.

The highest frequencies of resistance in isolates from pigs in both MSF and SSF were found against tetracycline. This is in accordance with earlier reports on the long term and extensive use of tetracycline that has resulted in a high prevalence of tetracycline-resistant bacteria in livestock [7,10,12,14,20]. Additionally, the prefabricated feed concentrate used in some SSFs and MSFs may have contained tetracyclines without our or the farmers’ knowledge due to poor labelling. Even so, it should be noted that the resistance frequency to tetracycline was significantly higher in the SSF than in the MSF. We cannot find a plausible explanation for this difference, as just one SSF was reported to have used tetracyclines by injection and none of the MSFs did [21]. However, given the overall high frequency of resistance, the usefulness of tetracycline as a veterinary drug can be strongly questioned. The high frequency of resistance to chloramphenicol in the pig isolates, regardless of farm type, is alarming, as this is an antibiotic highly important for human medicine, as are the somewhat lower, but still high, frequencies of resistance to the critically important antibiotics, ciprofloxacin and gentamicin [11]. Resistance to chloramphenicol, trimethoprim/sulfamethoxazole and gentamicin were more common in isolates from MSF compared with isolates from SSF. This is in line with the generally higher frequency of resistance found in intensified medium or large-scale farms in Thailand [10,22].

Over the last 15 years, there has been a rapid increase in livestock-associated MRSA (LA-MRSA) in domestic animal populations globally, especially in pigs [23]. Livestock-associated MRSA has become an important zoonotic agent, mainly affecting persons with direct animal contact. In this study we were not able to detect MRSA in any of the investigated pig farms, indicating a fortunate low prevalence in the sampled area.

In the present study, high tetracycline resistance detection rates were also found in *E. coli* isolates from contact, as well as non-contact, humans. These rates are about half the rates previously reported among human subjects in Thailand [24,25]. Still, one can speculate about a spread of tetracycline-resistant bacteria from pigs to contact humans to non-contact humans, as this resistance is often plasmid-mediated with a wide variety of genetic determinants [26].

Lower resistance detection rates were found for cefotaxime and gentamicin, whilst no resistance to meropenem was found in our isolate collection. These results are consistent with the association of antibiotic resistance to antibiotic use [2]. In our study, only a limited number of isolates were resistant to third generation cephalosporins, but resistance detection rates to fluoroquinolones were relatively high in both pigs and humans. This is in accordance with previous reports from the same region [10]. Fluoroquinolones are one of the most valuable antibiotic classes for the treatment of human infections and many countries have reduced their use in food-producing animals [2,27]. Thus, the careful monitoring of resistance to these agents is needed, especially among animals sent for slaughter and subsequently reaching the consumers.

We found a higher frequency of resistance to some critical and highly important antibiotics for human medicine in the MSFs compared with the SSFs. This is very alarming. Whether the resistance situation will be worsened when more SSFs are transformed to intensive MSFs, or large-scale farming, is hard to judge. The development is likely very much dependent on which and how antibiotics are used, and on the overall volume of antibiotics used.

## 4. Materials and Methods

### 4.1. Study Area and Study Population

The study was conducted on pig farms with sows in the Khon Kaen province, north-eastern Thailand 24 September to 22 December 2018. The inclusion criteria for the SSFs were that they should have 1–50 sows and for the medium-scale farms 100–500 sows, according to previous categorization in the country [20]. Within this sampling-frame, 51 MSFs and 113 SSFs were available for sampling in the study period. Details about the farm characteristics are described elsewhere [21]. In brief, none of the SSFs used antibiotics for disease preventive measures nor were they reported to give medication via feed. The MSFs applied a standardized animal health scheme where antibiotics were used regularly for disease prevention by injection and some farms also in medicated feed.

The SSFs were randomly selected from a list provided by the province veterinary service officer and then contacted to confirm that they were still keeping pigs during the planned fieldwork period. The villages with positive responses were visited and all farms where the farmers were at home agreed to participate. For the MSFs, the company officers were asked to select farms to be visited so the chosen farms were equally distributed over the districts in the province. This approach reduced the likelihood for a selection-bias towards MSFs with certain animal health statuses.

At each SSF and MSF, one farmer working at the pig farms (contact humans) and one who lived in the same household but without contact with the pigs (non-contact humans) were approached for voluntarily sampling. At some farms, only contact or non-contact humans were available, resulting in a total of 158 contact human samples and 114 non-contact human samples from the 164 pig farms included in the study. For details about the distribution of the samples, please see Supplementary Materials Table S1.

### 4.2. Collection of Fecal Samples for Escherichia coli Analyses

Individual rectal swab samples were only taken from healthy sows. In SSF farms with less than 10 sows, all sows at the farm were sampled, whereas in the other farms, 10 sows were randomly selected for sampling regardless of farm-type. In the SSFs, sampling was performed by a veterinarian, and at the MSFs by local veterinary assistants who were thoroughly instructed beforehand on how to collect the samples. In total, 839 pig fecal samples were obtained. The swabs were put in Amie’s charcoal transport medium tubes (Deltalab, Barcelona, Spain). Human freshly voided fecal samples were collected at the farms from one contact human and one non-contact human. In total, 272 human fecal samples were obtained. The samples were placed on ice and transported back to the laboratory at the Faculty of veterinary medicine, Khon Kaen University within 8 h. They were stored for a maximum of 48 h at 2–4 °C until subsequent analysis.

### 4.3. Collection of Swab Samples for Methicillin Resistant Staphylococcus Aureus (MRSA) Detection

MRSA samples from animals and farm environment were collected as previously described [28] using sterile swab cloths soaked in sterile saline (Sodibox™, Pont C’hoat, Nevez, France). Animal samples were collected by rubbing the skin behind ears. One cloth was used to sample approximately 20 pigs in MSFs. In SSFs, up to 20 animals were sampled per cloth, always including all sows in the herd. Environmental samples were also collected on each farm by rubbing cloths at selected contact points (up to 15 contact points on each cloth). From each SSF, one animal and one environmental sample were collected. From the MSF, a total of five samples were taken; three from animals and two from the environment. MRSA samples from humans were obtained by sampling the nares using swabs from DeltaLab. Swab samples were collected from one contact human and from one non-contact human on each farm.

### 4.4. Isolation of Escherichia coli

From each sample, fecal material was directly streaked onto MacConkey agar (Oxoid, Hampshire, England) and incubated at 37 °C overnight. Lactose-fermenting colonies with typical *E. coli* morphology were randomly selected. These isolates were sub-cultured onto blood agar, incubated overnight at 37 °C and tested for tryptophanase (indole) production using Motility-Indole-Lysine (MIL) medium (Oxoid). One indole positive isolate was selected from each fecal sample for further analyses and transferred to 1.8-mL cryotube (Nunc, Numbrecht, Germany) containing brain heart infusion broth (BHI, Oxoid) with 15% glycerol and stored at −80 °C. The isolates were transported frozen to the Swedish University of Agricultural Sciences (SLU), Uppsala, Sweden. The species identity of each bacterial isolate as *E. coli* was confirmed using matrix-assisted laser-desorption/ionization time-of-flight mass spectrometry (MALDI-TOF MS, Bruker Daltonik, Bremen, Germany).

### 4.5. Laboratory Analyses for Methicillin-Resistant Staphylococcus Aureus (MRSA)

Animal and farm environmental samples (cloths) were transferred to a plastic beaker and 200–400 mL Mueller Hinton broth with 6.5% NaCl was added. Cloths from medium sized herds were pooled; the two environmental cloths were pooled together, and the three animal samples were pooled together. Cloths from SSFs were analyzed individually. Swabs from humans were cut into falcon tubes and 5 mL of Mueller Hinton broth with 6.5% NaCl was added. The samples were incubated at 37 °C for 18–24 h. After incubation 10 μL were plated on Brilliance MRSA 2 agar (Oxoid) and incubated at 37 ± 1 °C for 24 ± 2 h. Suspected colonies were sub-cultured on blood agar plates and subjected to further analysis. For system control, MRSA (ATCC 35603) and methicillin sensitive *S. aureus* (ATCC 25923) were included in each setup. Presumptive MRSA isolates were confirmed by PCR targeting the genes *mecA, mecC* and *nuc*. Previously published PCR methods were used [29,30]. Positive and negative controls were included in each setup.

### 4.6. Antimicrobial Susceptibility Testing

Susceptibility testing of *E. coli* isolates to a panel of antimicrobial agents was carried out using the Kirby–Bauer method (disc diffusion method) following the guidelines given by The European Committee on Antimicrobial Susceptibility Testing [31]. The inhibition zones were interpreted using clinical breakpoints from [31]. The antibiotics in the test panel were chosen based on what vets and pharmacies said were the most commonly used for pigs and humans, respectively, in the region and then by representing different classes of antibiotics. The test panel included cefotaxime (5 μg), ciprofloxacin (5 μg), gentamicin (10 μg), chloramphenicol (30 μg), meropenem (10 μg), tetracycline (30 μg) and trimethoprim/sulfamethoxazole (1.25/23.75 μg). *E. coli* CCUG 17620 was used as an antimicrobial-susceptible control strain on a daily basis. Multidrug resistance was defined as isolates resistant to three or more classes of antimicrobial agents.

### 4.7. Data Analysis

Univariate comparison of the resistance rate to the seven antibiotics and MDR among the varies categories of isolates—i.e., from SSF and MSF sows, SSF and MSF human (contact and non-contact)—was performed by Chi-square tests with the STATA statistical package v.15.0 (Stata Corporation, College Station, TX, USA). Pairwise analysis of correlation between the resistance to the different antibiotics was investigated using Pearson’s correlation analysis. A *P* value of <0.05 was considered significant.

All antimicrobial susceptibility testing results were compiled into one table (Supplemental Table S1). An overview heatmap was generated for the antimicrobial susceptibility results for each bacterial isolate to the seven antimicrobial agents (Supplemental Figure S1) using a customized python script. In the same script, an additional bar chart was generated displaying the percentage of isolates in each group based on isolate source and farm size and of all antibiograms present (Supplemental Figure S2).

### 4.8. Ethical Approval

The study was conducted according to the Helsinki declaration for the human subjects and the EU Directive 2010/63/EU for animal experiments; the protocol involving human participants and animals was approved by the Khon Kaen University Ethics Committee (Project ID: HE612268 and 0514.1.75/66, respectively). Informed consent was obtained from each human subject after the experimental procedures had been completely explained. Pig samples were collected at the farms with the permission of the owner of the pig herd.

## 5. Conclusions

Our hypothesis that the antibiotic resistance situation was worse in intensive pig farming compared with small-scale farming was supported by our findings of higher frequency of resistance to some critical and highly important antibiotics for human medicine in pig isolates from the MSFs compared to the SSFs. On the other hand, our findings do not support the hypothesis that farmers working at the MSFs had a higher rate of AMR in their fecal *E. coli* compared with those working at the SSFs. Overall, the current resistance situation in the two pig farming systems in the region is unsatisfactory from an animal health perspective where several antibiotics are not functioning as expected, as well as from the public health perspective, given the risk of transmission to humans. The frequency of resistant bacteria in pigs, as well as humans, to several of the antibiotics tested for was very high compared to several European countries, but similar to others in SE Asia. This calls for a reduction in antimicrobial use in livestock as well in humans for mitigating the global AMR emergence.

## Figures and Tables

**Table 1:** Percentages and numbers of *E. coli* isolates from pigs and contact- and non-contact humans exhibiting resistance to various antimicrobial agents at medium-scale farms (MSFs) and small-scale farms (SSFs) in North-Eastern Thailand.

Antimicrobial Agents	Resistant Isolates % (n)						
	Pigs			Humans			Overall (*n* = 987)
	MSF (*n* = 457)	SSF (*n* = 300)	Total (*n* = 757)	Contact (*n* = 139)	Non-Contact (*n* = 91)	Total (*n* = 230)	
Cefotaxime	0.9 (4)	1.0 (3)	0.92 (7)	2.2 (3)	2.2 (2)	2.2 (5)	1.2 (12)
Chloramphenicol	42.7 (195) a	26.0 (78) a	36.1 (273) d	15.1 (21)	13.2 (12)	14.3 (33) d	31.0 (306)
Ciprofloxacin	13.8 (63)	11.7 (35)	12.9 (98)	10.8 (15)	14.3 (13)	12.2 (28)	12.8 (126)
Gentamicin	6.8 (31) b	4.3 (13) b	5.8 (44)	3.6 (5)	4.4 (4)	3.9 (9)	5.4 (53)
Meropenem	0 (0)	0 (0)	0 (0)	0 (0)	0 (0)	0 (0)	0 (0)
Tetracycline	52.9 (242) c	64.7 (194) c	57.6 (436) d	38.4 (54)	48.4 (44)	43.0 (99) d	54.2 (535)
Trimethoprim/Sulfamethoxazole	46.4 (212) a	33.0 (99) a	41.1 (311) d	21.6 (30)	22.0 (20)	21.7 (50) d	36.6 (361)
Multidrug-resistant	22.0 (128)	28.0 (66)	25.6(194) c	12.2 (17)	13.2 (12)	12.6 (29) c	22.6 (223)

Percentages on the same row with same letter are significantly different from each other: a = *P* < 0.001; b = *P* < 0.05; c = *P* < 0.01; d = *P* < 0.001.

**Table 2:** Percentages and numbers of *E. coli* isolates resistant to antibiotics from farmworkers handling pigs (contact humans) in small- and medium-scaled farms in North-Eastern Thailand.

	Resistant Isolates % (*n*)		
Antibiotic	Working in Small Scale Farms *n* = 92	Working in Medium Scale Farms *n* = 47	All Contact Humans *n* = 139
Cefotaxime	1.4 (2)	2.1 (1)	2.2(3)
Chloramphenicol	17.4 (16)	10.6 (5)	15.1 (21)
Ciprofloxacin	11.9 (11)	8.5 (4)	10.8 (15)
Gentamicin	3.2 (3)	4.2 (2)	3.6 (5)
Meropenem	0 (0)	0 (0)	0 (0)
Tetracycline	39.1 (36)	38.3 (18)	38.8 (54)
Trimethoprim/Sulfamethoxazole	17.5 (16)	29.9 (14)	21.6 (30)
Multidrug-resistant	13.0 (12)	10.6 (5)	12.2 (17)

No significant difference in percentage observed between small- and medium-scale farms.

**Table 3:** Ten most common antibiograms of *E. coli* isolates from pigs and contact and non-contact humans at small- and medium-scale farms in North eastern Thailand.

Resistance Patterns	Percent (*n*) of Isolates with the Respective Antibiogram				
	Pigs		Humans		Total (*n* = 987)
	Medium-Scale (*n* = 457)	Small-Scale (*n*= 300)	Contact (*n* = 139)	Non-Contact (*n* = 91)	
TET	15.3 (70)	29.7 (89)	14.4 (20)	31.2 (19)	20.0 (198)
CHL-SXT-TET	18.8 (86)	15.7 (47)	5.8 (8)	6.6 (4)	14.7 (145)
CHL-SXT	11.2 (51)	2.3 (7)	1.4 (2)	0.0 (0)	6.8 (60)
SXT-TET	4.8 (20)	6.7 (20)	5.0 (7)	9.8 (6)	5.4 (53)
CHL-TET	3.9 (18)	2.0 (6)	5.0 (7)	3.3 (2)	3.3 (33)
CIP-TET	2.2 (10)	4.3 (13)	2.9 (4)	6.6 (4)	3.1 (31)
SXT	3.9 (18)	1.3 (4)	2.9 (4)	4.9 (3)	2.9 (29)
CIP	2.8 (13)	2.0 (6)	0.7 (1)	1.6 (1)	2.1 (21)
CHL	2.0 (9)	2.3 (7)	0.0 (0)	1.6 (1)	1.7 (17)
CHL-CIP-SXT-TET	1.8 (8)	2.0 (6)	0.7 (1)	3.2 (2)	1.7 (17)

CHL, chloramphenicol; CIP, ciprofloxacin; SXT, trimetroprim/sulfametoxazole; TET, tetracycline.

**Table 4:** Pairwise correlation between two antimicrobial resistance statuses in *E. coli* isolates collected at small- and medium-scale farms in North Eastern Thailand.

Antimicrobial Agent Resistance Combination		Correlation
Chloramphenicol	Trimethoprim/Sulfamethoxazole	**0.60 ^1^**
Cefotaxime	Gentamicin	**0.26**
Trimethoprim/Sulfamethoxazole	Tetracycline	**0.24**
Ciprofloxacin	Gentamicin	**0.23**
Chloramphenicol	Tetracycline	**0.23**
Ciprofloxacin	Cefotaxime	**0.15**
Gentamicin	Tetracycline	**0.12**
Ciprofloxacin	Tetracycline	0.09
Gentamicin	Trimethoprim/Sulfamethoxazole	0.07
Ciprofloxacin	Trimethoprim/Sulfamethoxazole	0.07
Chloramphenicol	Cefotaxime	0.05
Chloramphenicol	Gentamicin	0.03
Cefotaxime	Trimethoprim/Sulfamethoxazole	0.03
Cefotaxime	Tetracycline	0.03
Chloramphenicol	Ciprofloxacin	0.03
Multidrug-resistant	Chloramphenicol	**0.65**
	Trimethoprim/Sulfamethoxazole	**0.64**
	Tetracycline	**0.45**
	Gentamicin	**0.26**
	Ciprofloxacin	**0.25**
	Cefotaxime	**0.14**

^1^ Numbers in boldface represent statistically significant (*P* < 0.05) correlations.

## Supplementary Materials

The following are available online at https://www.mdpi.com/2079-6382/9/10/662/s1, Figure S1: Susceptibility Heat map; Figure S2: Antibiogram; Table S1: Susceptibility test.
