# Supplementary material for: Antimicrobial Resistance in Fecal Escherichia coli from Humans and Pigs at Farms at Different Levels of Intensification
Source: Antibiotics (Basel). 2020 Sep 30;9(10):662. doi: 10.3390/antibiotics9100662 (PMC7650604; doi:10.3390/antibiotics9100662)
Supplement: Supplementary file 1 [file antibiotics-09-00662-s001.zip › Supplemental_Figures 1 and 2.docx..docx]

**
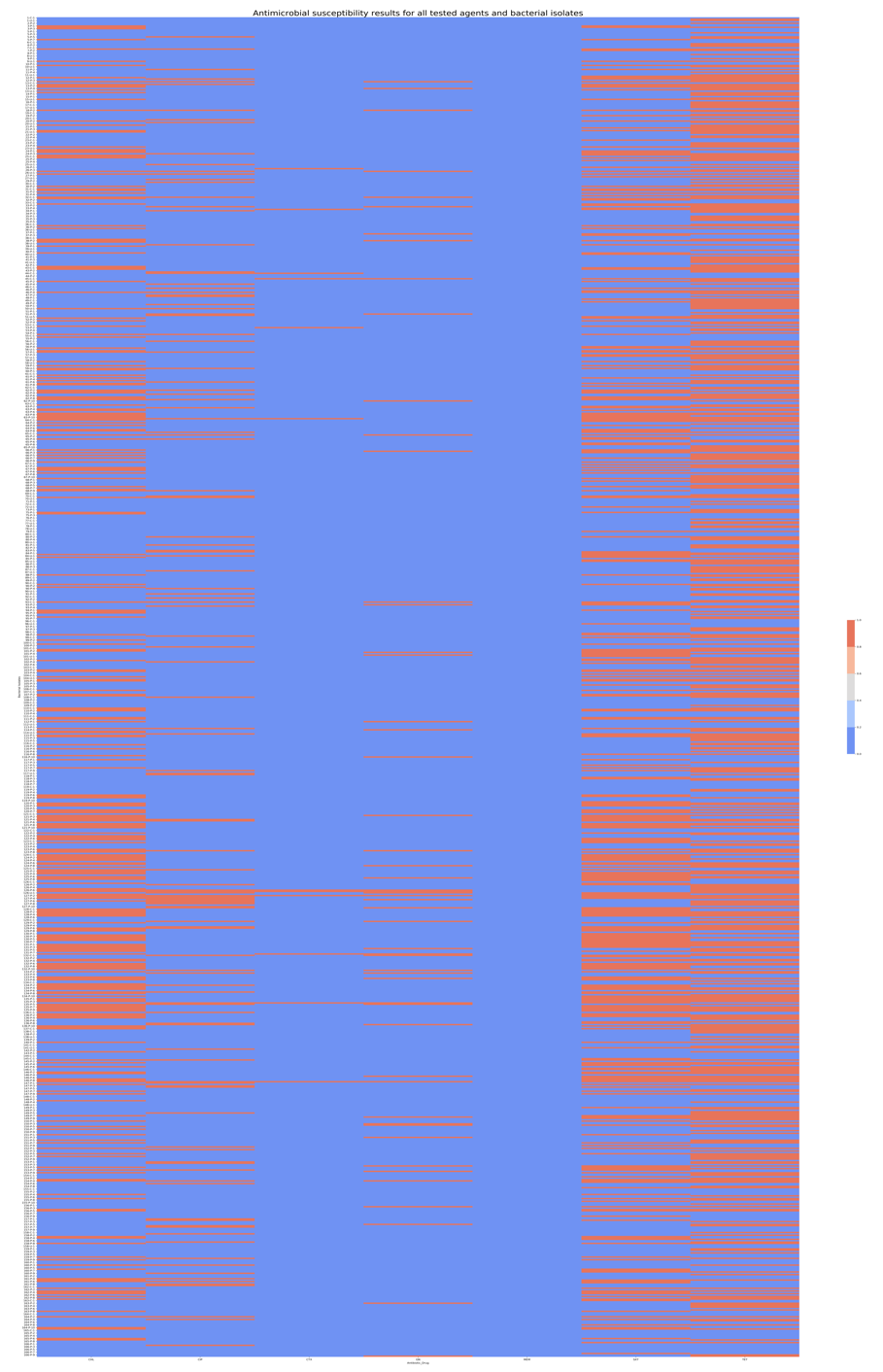
**

**Supplemental Figure 1** Heatmap demonstrating the distribution of phenotypic resistance to seven clinical antimicrobial agents among all *E. coli* isolates, where drug susceptibility is blue with a resistance value of 0 and drug resistance is red with a resistance value of 1.


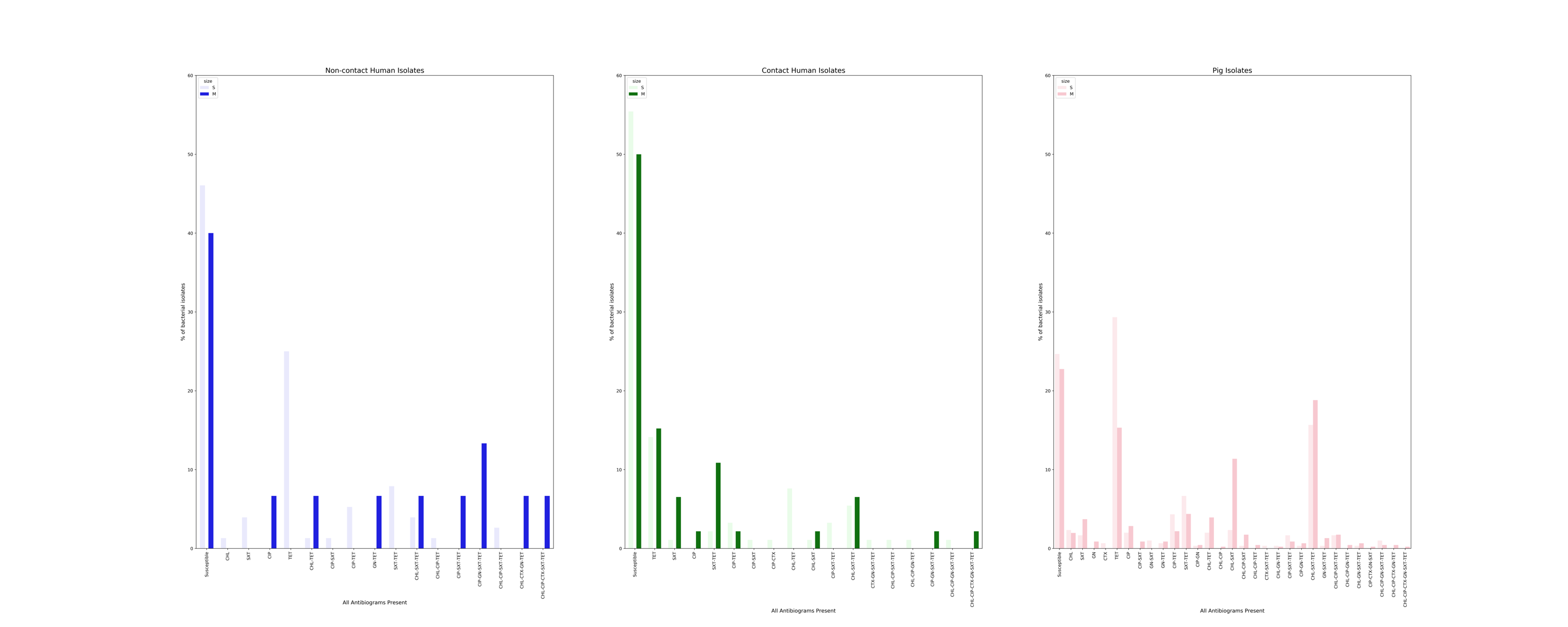


**Supplemental Figure 2** Bar chart showing the % distribution of phenotypic antibiograms in each group based on farm size and host source of *E. coli* isolates.
